# Research on the data validity of a coal mine solid backfill working face sensing system based on an improved transformer

**DOI:** 10.1038/s41598-023-38365-6

**Published:** 2023-07-08

**Authors:** Lei Bo, Shangqing Yang, Yang Liu, Yanwen Wang, Zihang Zhang

**Affiliations:** School of Mechanical Electronic and Information Engineering, China University of Mining and Technology (Beijing), Beijing, 100083 China

**Keywords:** Coal, Engineering, Computer science, Information technology

## Abstract

Solid backfilling in coal mining refers to filling the goaf with solid materials to form a support structure, ensuring safety in the ground and upper mining areas. This mining method maximizes coal production and addresses environmental requirements. However, in traditional backfill mining, challenges exist, such as limited perception variables, independent sensing devices, insufficient sensing data, and data isolation. These issues hinder the real-time monitoring of backfilling operations and limit intelligent process development. This paper proposes a perception network framework specifically designed for key data in solid backfilling operations to address these challenges. Specifically, it analyses critical perception objects in the backfilling process and proposes a perception network and functional framework for the coal mine backfilling Internet of Things (IoT). These frameworks facilitate rapidly concentrating key perception data into a unified data centre. Subsequently, the paper investigates the assurance of data validity in the perception system of the solid backfilling operation within this framework. Specifically, it considers potential data anomalies that may arise from the rapid data concentration in the perception network. To mitigate this issue, a transformer-based anomaly detection model is proposed, which filters out data that does not reflect the true state of perception objects in solid backfilling operations. Finally, experimental design and validation are conducted. The experimental results demonstrate that the proposed anomaly detection model achieves an accuracy of 90%, indicating its effective detection capability. Moreover, the model exhibits good generalization ability, making it suitable for monitoring data validity in scenarios involving increased perception objects in solid backfilling perception systems.

## Introduction

Solid backfill technology refers to filling the extraction area with solid material to form a support body to secure surface and upper mining areas, a method designed to provide support and stabilize the geological conditions of the mining area. Solid filling helps to reduce surface subsidence, reduce the environmental impact of coal gangue and create a safer and more efficient mining environment^1^. It is an effective mining waste treatment and green mining technology^2^. With the continuous growth in the world’s population and industrialization, the energy demand is also increasing. Coal, as an important energy resource, occupies an important position in world energy consumption^3^. Taking data from China’s National Bureau of Statistics as an example, in 2020, China’s crude coal production reached 2.76 billion tonnes, accounting for 67.6% of the total primary energy production, and coal consumption reached 2.83 billion tonnes, accounting for 56.8% of the total energy consumption^4^. In the medium and long term, coal is still the energy backbone of China’s economy and plays an extremely important role in the country’s economic development.

However, traditional coal mining methods often lead to a series of environmental problems^5^, such as land destruction, water pollution and air pollution^6^. These problems not only harm the human living environment but also pose challenges to the sustainable development of the coal industry. The amount of coal mined from the “three-underground” (under buildings, railways and water) in China is more than 14 billion tons^7^, and the “three-underground” coal mined accounts for only approximately 7% of the total coal mining. Due to the influence of deep coal seams^8^, China’s coal mining mainly uses underground mining methods^9^. After the coal is extracted, a goaf is formed at the original location. Under the action of the overlying strata, the upper strata of the goaf will collapse, fracture, bend and sink causing rock falls such as coal roof falls and ground collapses^10^. Data statistics show that the cumulative economic loss caused by coal mining-induced goaf collapse in China has exceeded 50 billion yuan, and the per capita area of goaf collapse in mining areas also exceeds 1.8 $\hbox {hm}^2$^11^.

The ecological damage caused by coal mining, goaf damage causing mining accidents and geological disasters such as unstable waste dumps triggering landslides seriously threaten the safety and development of the energy industry. To solve these problems, coal mining needs to transition to environmental protection and sustainable development, and applying solid backfill technology is important to this transition process^12^. Solid backfill mining is filling mined-out goaf areas with solid waste to constrain overburden strata movement and avoid surface subsidence, thus protecting surface infrastructure by controlling surface subsidence. It is an important technical approach to solving the “three-unders” coal compaction problem, coal gangue emissions, and land resource problems, reducing mining areas, extending the life of mines, improving the recovery rate of mineral resources, reducing environmental pollution, achieving green mine construction and promoting the sustainable development of coal mining. It has significant economic, social and environmental benefits.

Currently, large-scale smart mines in China have entered a period of rapid development. The development of unmanned and green mining has become an important method for achieving harmonious coexistence between man and nature in the new era^13^. As a key component of green mining construction, solid backfilling has received increasing attention.

Li et al.^14^ examined the current state of development of intelligent mining faces for fully mechanized mining, both domestically and internationally. There are still five major problems that need to be solved to achieve intelligence: the low level of equipment intelligence, low reliability of building a refined geological model, unresolved automatic detection and control of the “three flats and two straights” of the face, immature technology of automatic movement of advanced support in the roadway, and lack of a breakthrough in the identification of coal and gangue in the fully mechanized face. Based on the Industrial Internet of Things technology, a mining face perception system was tentatively established, and the key technologies for intelligent control were discussed.

Zhang et al.^15^ built an intelligent mining big data analysis and decision-making platform for the mining face establishing a monitoring system to solve the problem of intelligent analysis and decision-making in the mining process.

Huang et al.^16^ applied intelligent backfilling technology in the Tiaoshuihe phosphate mine, which not only ensured and improved the production capacity of the backfilling system but also further reduced the backfilling cost. Although fruitful research on the intelligent system coal mining method has preliminarily applied intelligent methods to the gangue ratio, the backfilling effect is still mainly based on human judgement. There has not been much research on multisource sensor data fusion and reconstruction methods after establishing a perception system. Therefore, to achieve intelligent solid backfilling, the backfilling perception and data effectiveness problems must still be solved^17^.

In intelligent backfilling mining, collecting sensor information is an important step to achieving intelligent filling. The filling system needs to perceive the underground environment in real time, such as the filling angle, working face inclination angle and filling speed parameters, for judging the operation status and filling effect of the equipment, thus realizing intelligent and unmanned filling mining. Due to the harsh environment in underground coal mines where the filling working face is located, the sensor network inevitably produces abnormal data significantly different from normal ranges. These abnormal data cannot objectively reflect the underground environment and working face operating conditions. Thus, they need to be removed promptly to ensure that correct decisions are made in subsequent application layers.

There are many different types of multisource heterogeneous sensors in the filling workspace. They detect changes in different objects. For example, angle sensors are used to measure the tilt angle of hydraulic supports, while distance sensors are used to measure the material height. Although these sensors sense different objects, they all belong to one system; therefore, there is a potential correlation between different sensor data. When performing anomaly detection, it is necessary to fully consider and utilize this potential correlation. The transformer model is a typical end-to-end model proposed in 2017^18^.

In previous end-to-end models, recurrent neural networks (RNNs) or convolutional neural networks (CNNs) were commonly used. These models process only one element sequentially, not all elements in parallel. In contrast, a transformer is an end-to-end model based on an encoder–decoder framework that does not use RNNs or CNNs but fully exploits attention mechanisms. By stacking multiple encoders and decoders, the transformer has achieved better performance in discovering long-term historical sequence dependencies. Due to its outstanding performance, researchers have gradually applied it to time series modelling and prediction and proposed various variant models based on transformers.

Cai et al.^19^ proposes the traffic transformer model by combining a transformer with a graph convolutional network (GCN) neural network for traffic flow prediction, which achieves better performance than baseline models on traffic datasets. Lin et al.^20^ combines a transformer with a state space model to propose the SSDNet model for solar energy and power data prediction. Tuli et al.^21^ applies adaptive and adversarial learning mechanisms to a transformer to propose the TranAD model for anomaly detection, achieving excellent results on several industrial datasets. Wang et al.^22^ combines a residual variational autoencoder structure with a transformer for anomaly detection in satellite remote sensing data. The temporal fusion transformers model is proposed by Lim et al.^23^, used for multivariable time series prediction problems. The model adds multiple variable selection networks based on transformer-based models, which can better predict multivariable time series and achieve superior performance on datasets such as the electricity industry, retail industry, and traffic flow. The self-attention mechanism of a transformer has been preliminarily applied to predict and detect anomalies using potential correlations. However, although the parallel processing method of the self-attention mechanism improves the ability of models to capture long-term historical dependencies, it also makes it difficult for models to effectively extract temporal information from sensor data sequences. Therefore, it is necessary to perform position encoding before inputting sensor data into the model so that temporal information becomes independent feature variables. In addition, considering that the data generated by a multisensor network in face-fill mining is a typical multivariate time series dataset, predictive models need to fully consider the relationships between sequences during prediction by assigning different weights according to different situations and finding appropriate prediction error thresholds.

In response to the insufficient validity of solid backfilling perception data in coal mines, this article proposes a solid backfilling perception method based on data validity assurance. Through establishing a solid backfilling Internet of Things framework to enhance the perception ability of the filling environment and centralized data sharing, a transformer model-based abnormal detection model for solid backfilling perception data is constructed to improve data reliability. Finally, the effectiveness of the model proposed in this paper is verified through experiments.

## Objective of solid backfill perception

The factors influencing the backfilling effect can be divided into two categories: static factors and dynamic factors. Static factors include geological conditions, coal seam orientation, fill material ratio, gangue aggregate grading, compaction distance from the top and drop centre distance; dynamic factors include working face inclination angle, fill material moisture, material uniformity, fill body freedom degree, compaction force, compaction angle, spatiotemporal characteristics and compaction degree. Among them, other mining faces have made many relevant perceptions about geological conditions and coal seam orientation, which can be provided as data sharing to the backfill system. In addition to data sharing with other mining systems, it is also necessary to establish a dedicated perception system for the filling system. The objects perceived in the filling system are divided into two categories: perception of materials and perception of the filling action status.

Backfilling operations must account for various physical properties of the material to be filled, as well as environmental changes and dynamic actions during filling. A critical aspect is the filling process batching system, which detects and measures the proportioning of materials through speed measurement, weighing, and belt scale instruments. The main objects sensed are, therefore, related to feeding fillers, such as their proportion and quantity. To achieve this level of precision, sensing devices such as feeder belt speed sensors and weight sensors are included in the process.

**Figure 1:**
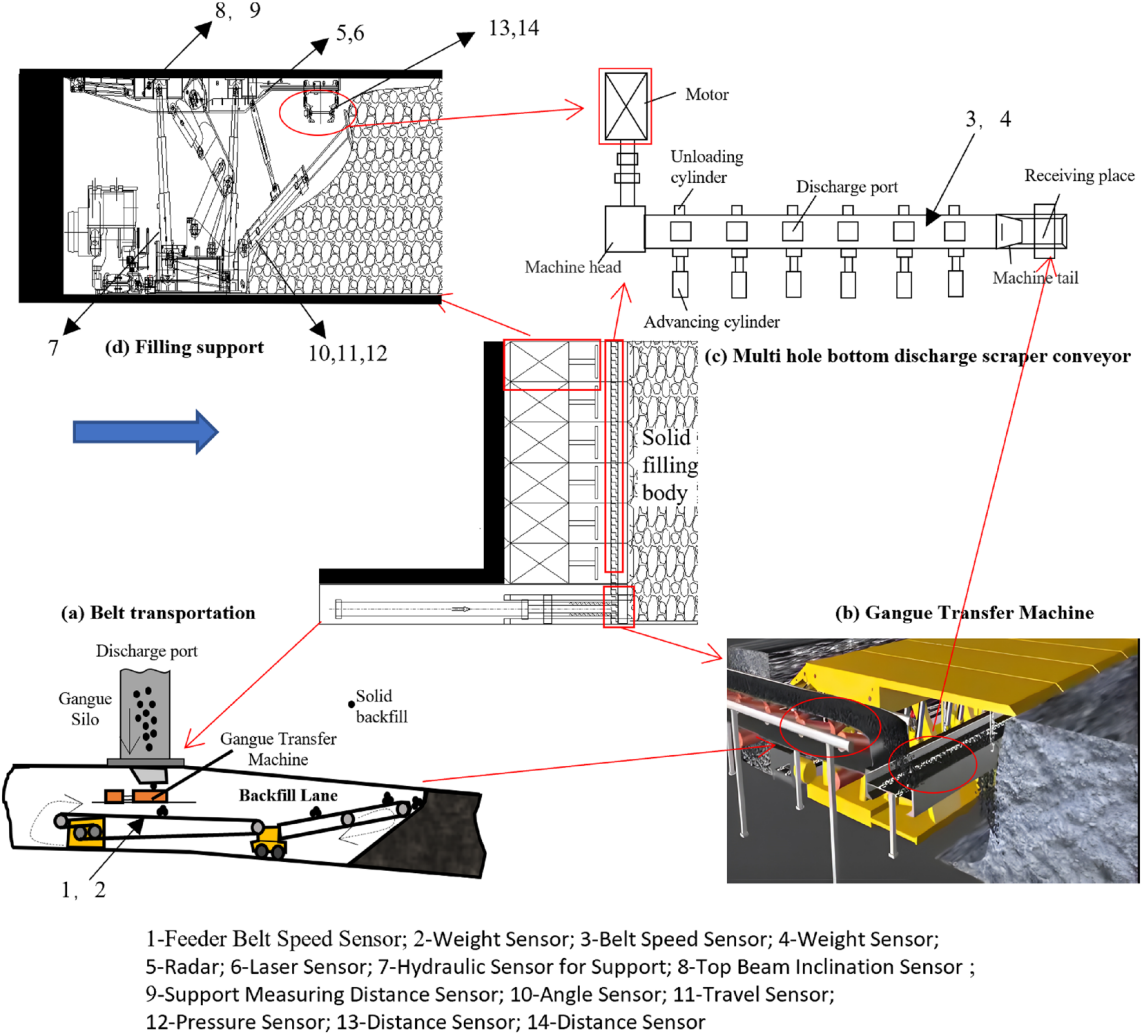
Filling the overall equipment transportation system and its layout diagram.

Figure 1 presents a cross-sectional view of the underground transportation process of filling materials. Waste rock transfer machine is displayed in figure (a), and figure (c) shows the porous bottom unloading filling scraper conveyor, which comprises discharge holes on each middle trough plate. The hydraulic jacks, located on both sides of the middle trough, control the opening and closing of the discharge holes. The scraper conveyor is positioned under the rear top plate of the filling hydraulic support and positioned in parallel to the filling working face. Figure (d) is a filling support. The transportation process of filling materials progresses through four stages. First, (a) the solid filling materials are fed into the underground through the feeding hole. Subsequently, the waste rock transfer machine is used to transport the filling materials from the roadway to the filling working face through belt transportation. The filling materials are lifted to the height where the porous bottom unloading filling scraper conveyor is in position. Then, (b) the filling materials are transferred from the waste rock transfer machine to the porous bottom unloading filling scraper conveyor. (c) Subsequently, the scraper conveyor is placed under the rear top plate of the filling hydraulic support and parallel to the filling working face. As per the requirement of filling discharge, the scraper conveyor opens the discharge port and discharges the filling materials behind the filling hydraulic support. (d) Finally, the compaction mechanism of the filling support compresses and compacts the filling materials into the filling area, creating a filler that fortifies the goaf’s roof. To ensure optimum performance, monitoring the infeed speed and height is important. The sensing equipment included in this process includes a conveyor speed sensor, weight sensor, end head height sensor, and scraper machine straightness (laser).

**Figure 2:**
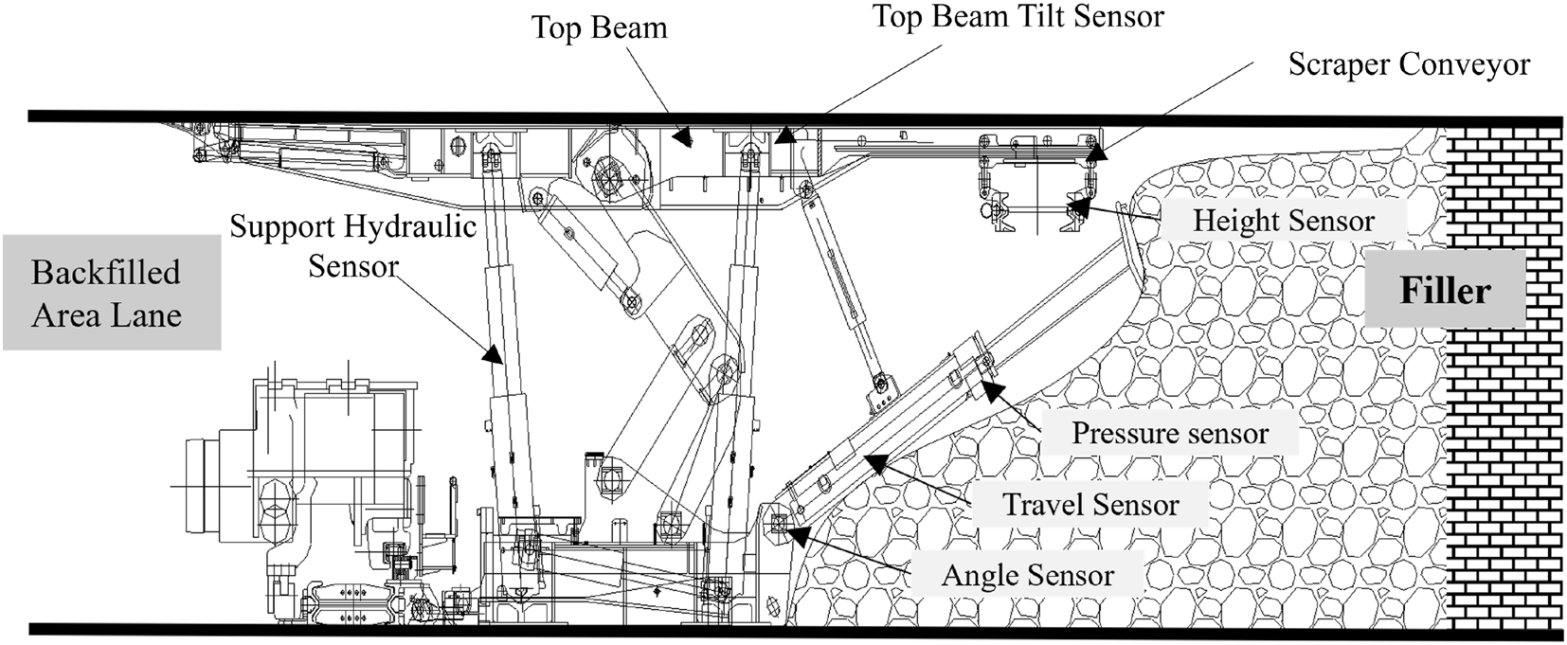
Schematic diagram of the sensor layout for filling equipment in the gangue filling working face.

Figure 2 shows a view of the face fill for solid backfill mining. This includes hydraulic support, scraper conveyors, throwing machines and their supporting equipment systems. The hydraulic support consists of structural components, jacks, hydraulic control valve groups, and piping. The jack controls the action of the hydraulic support. The key to the sensing system is sensor sensitivity and stability, while reliable operation depends on the sensor installation position. Objects sensed include support equipment such as hydraulic pressure and other support conditions, filling bracket action status, stacking materials, and filling status on a controlled work face. Hydraulic support sensors, top beam tilt sensors for hydraulic supports, height distance sensors for hydraulic supports, and ram horizontal and vertical height (angle, stroke, and pressure sensors) are used.

In summary, combined with the backfill workflow, the detection targets of the backfill detection system are shown in Table 1.

**Table 1:** Perception content statistics.

Perception system	Primary sensing device	Object of characterization
Filling material system	Feeder belt speed sensor	Material feeding speed
	Weight sensor	Material feeding weight
Filling material transport system	Belt speed sensor	Material discharge speed
	Weight sensor	Feeding amount and scraper conveyor Discharge amount
	Radar	Distance between discharge port and material pile
	Laser sensor	Levelness and smoothness of scraper
Filling face perception	Hydraulic sensor for support	Pressure of supporting equipment
	Top beam inclination sensor	Inclination angle of supporting tunnel
	Support measuring distance sensor	Height of supporting tunnel
	Angle sensor	Angle of filling push plate
	Travel sensor	Travel of filling push plate
	Pressure sensor	Pressure of filling push plate
	Distance sensor	Height of material pile
	Distance sensor	Horizontal distance between material Pile and filling mechanism

## Structural design of the Internet of Things perception system for solid backfilling in coal mines

Due to the limitations of underground coal mining, the availability of traditional mobile communication signals in solid backfill working conditions is reduced^24^. In addition, the informatization level is low, and monitoring data cannot be centrally viewed due to the dependence on different information technology manufacturers for various equipment and other issues. This results in a “data island” for solid backfill, leading to poor data availability for intelligent solid backfilling in coal mines. However, integrating intelligence and industry has created new opportunities for solid backfilling in coal mines^25^. Therefore, establishing internal IoT communication as a top priority task is crucial to providing data support for perception^26^.

**Figure 3:**
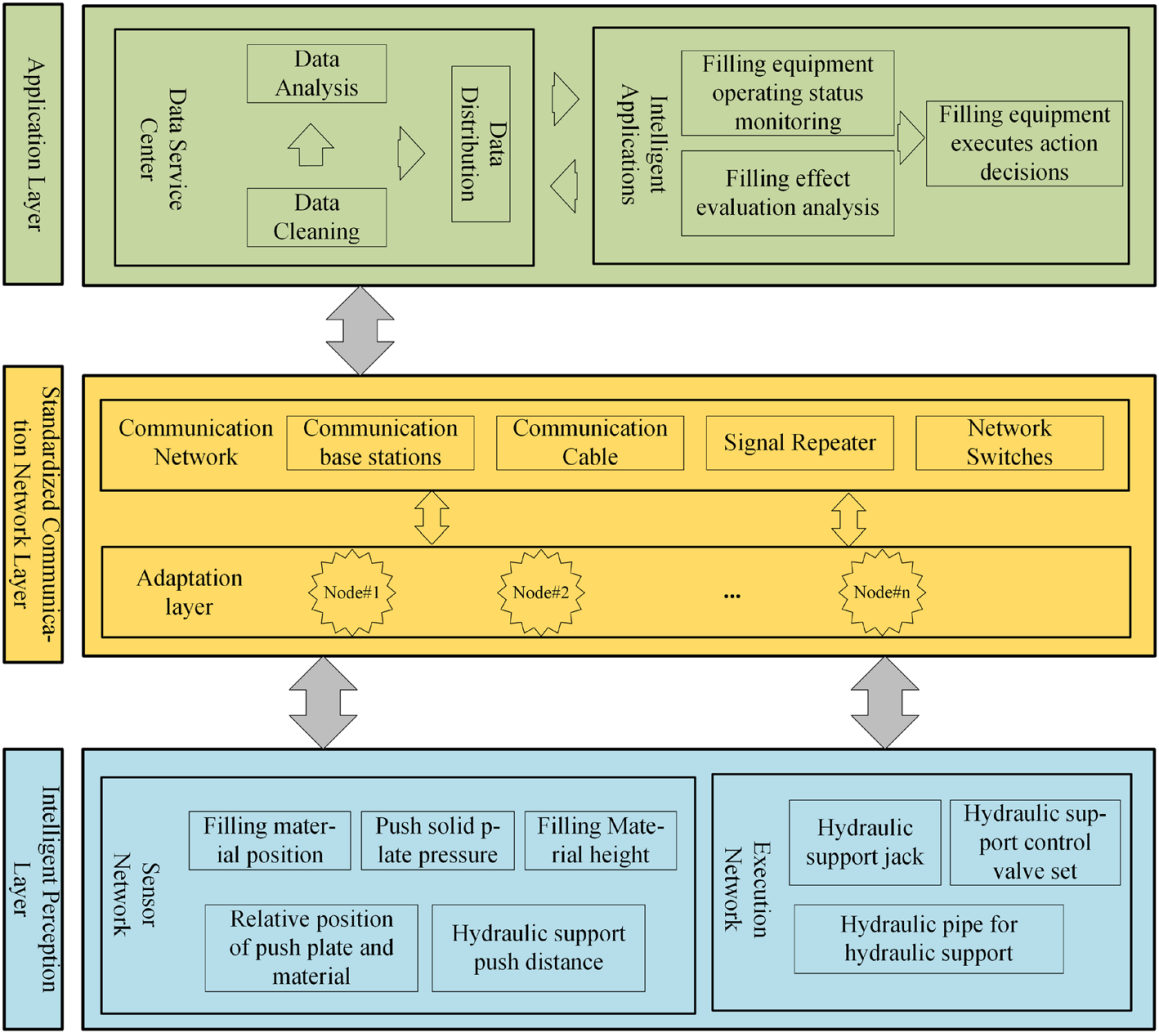
Solid backfill perception Internet of Things framework.

**Figure 4:**
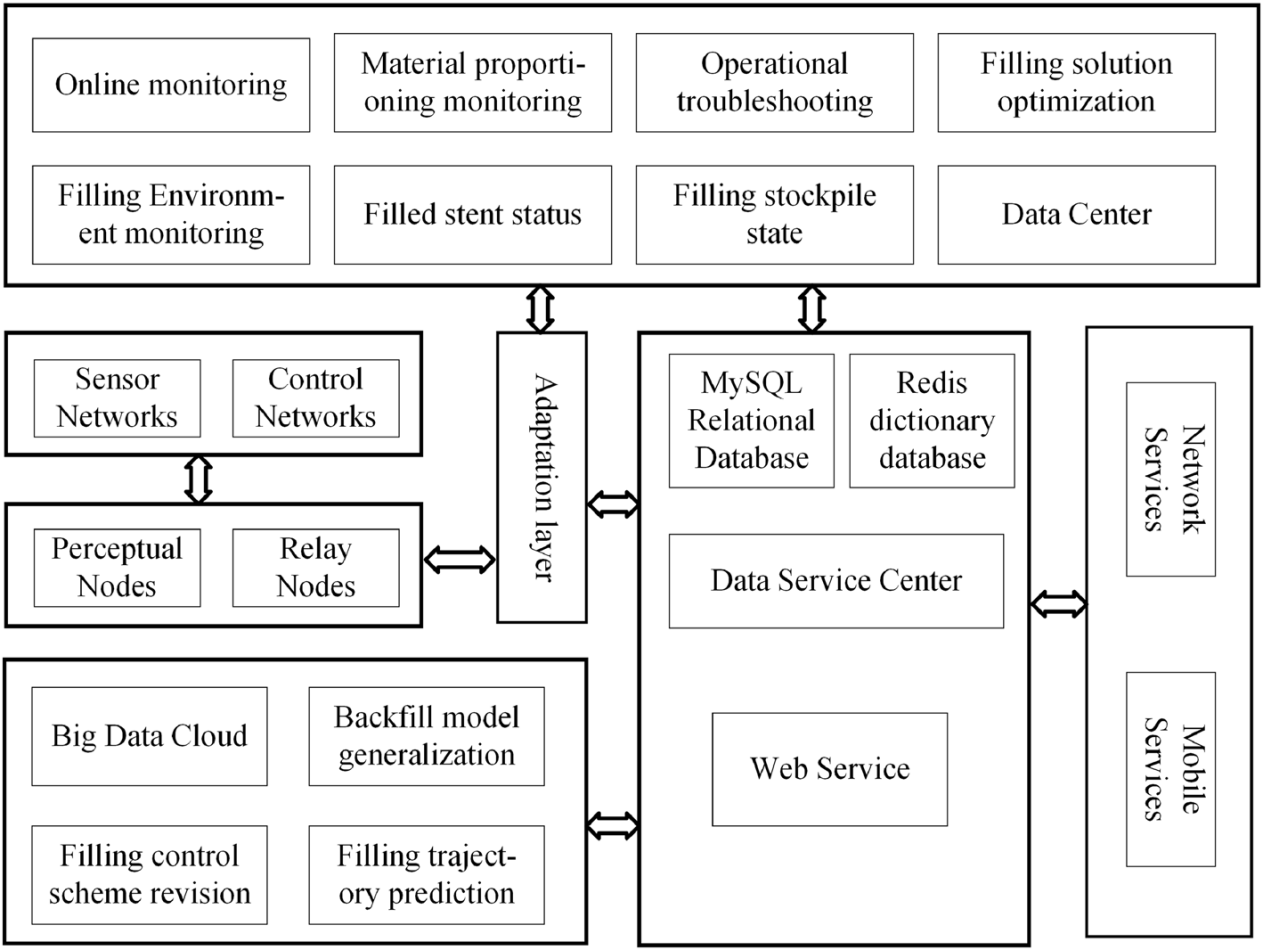
Application framework for solid backfill perception on the Internet of Things platform.

The intelligent filling and mining system consists of three functional parts: the intelligent perception layer, standardized communication network layer, and intelligent filling and mining application layer^26^. Figures 3 and 4 illustrate that the intelligent perception layer collects sensor and equipment status data. The collected data are then aggregated through the standardized communication network layer to the ground data service centre in the intelligent filling and mining application layer. From there, it is distributed to the corresponding application software for big data calculation and visualization monitoring. In addition, based on the results generated by the big data calculation, the ground control centres issue instructions to the filling equipment. These instructions are received, parsed, and executed by executing agencies in the intelligent perception layer through standardized communication network layers.

The intelligent perception layer consists of a sensor network and an execution network. It connects devices to the network, interconnecting people, devices and communication systems^27^. Each device in the intelligent perception layer has a unique node identifier in the communication system and an independent communication address after joining the network. It can establish stable communication with adapter nodes. The sensor network consists of sensors, such as pressure sensors, stroke sensors, tilt angle sensors, material level height sensors, and camera components. Among them, pressure sensors detect the force between the pusher mechanism and the filling material. Stroke sensors detect the degree of extension of the pusher mechanism. Tilt angle sensors detect the angle between the pusher and the hydraulic base. The material level height sensor detects the deformation height of the filling material. The video sensor detects the position status between the pusher and conveyor machine. The sensor network simultaneously uploads the real-time equipment status information collected by the abovementioned sensors along with environmental state information and filling effect information to the communication network layer. In contrast, the execution networks perceive the working states of the execution mechanisms for filling the mining faces, receive standardized downlink instructions from the communication network layers, parse according to system instructions, and execute filling actions.

The standardized communication network layer consists of adapter nodes and communication networks, which provide remote communication between work surface devices and control centres^28^. Among them, the adapter node is responsible for the communication function with hardware. It establishes a stable connection with multiple sensors or execution units through transmission control protocol (TCP) communication, forwards data to the server data centre upwards, and forwards application layer operation control instructions downwards. The communication network consists of communication base stations, cables, signal repeaters, and network switches to transmit data signals.

The application layer of intelligent filling and mining consists of a data service centre and intelligent applications^29^. The former is responsible for sharing the basic data uploaded by the intelligent perception layer, including data cleaning and distribution. Data cleaning is used to clean and optimize the received abnormal data. Data distribution sends data to related applications, such as distribution to visual monitoring applications for large-screen monitoring, to a data computing centre for data calculation, and remote servers for remote operation; the latter must be issued by the former when executing network send instructions sent by the intelligent perception layer. Intelligent applications are related applications that monitor the filling and mining equipment status, detect filling effects, and make action decisions. Based on monitoring data, geological environment maps of working faces are generated in real time at control centres. Based on big data calculations of fill effects, action instructions for next-step fill execution are generated, which are then issued to execution units by the data centre.

**Figure 5:**
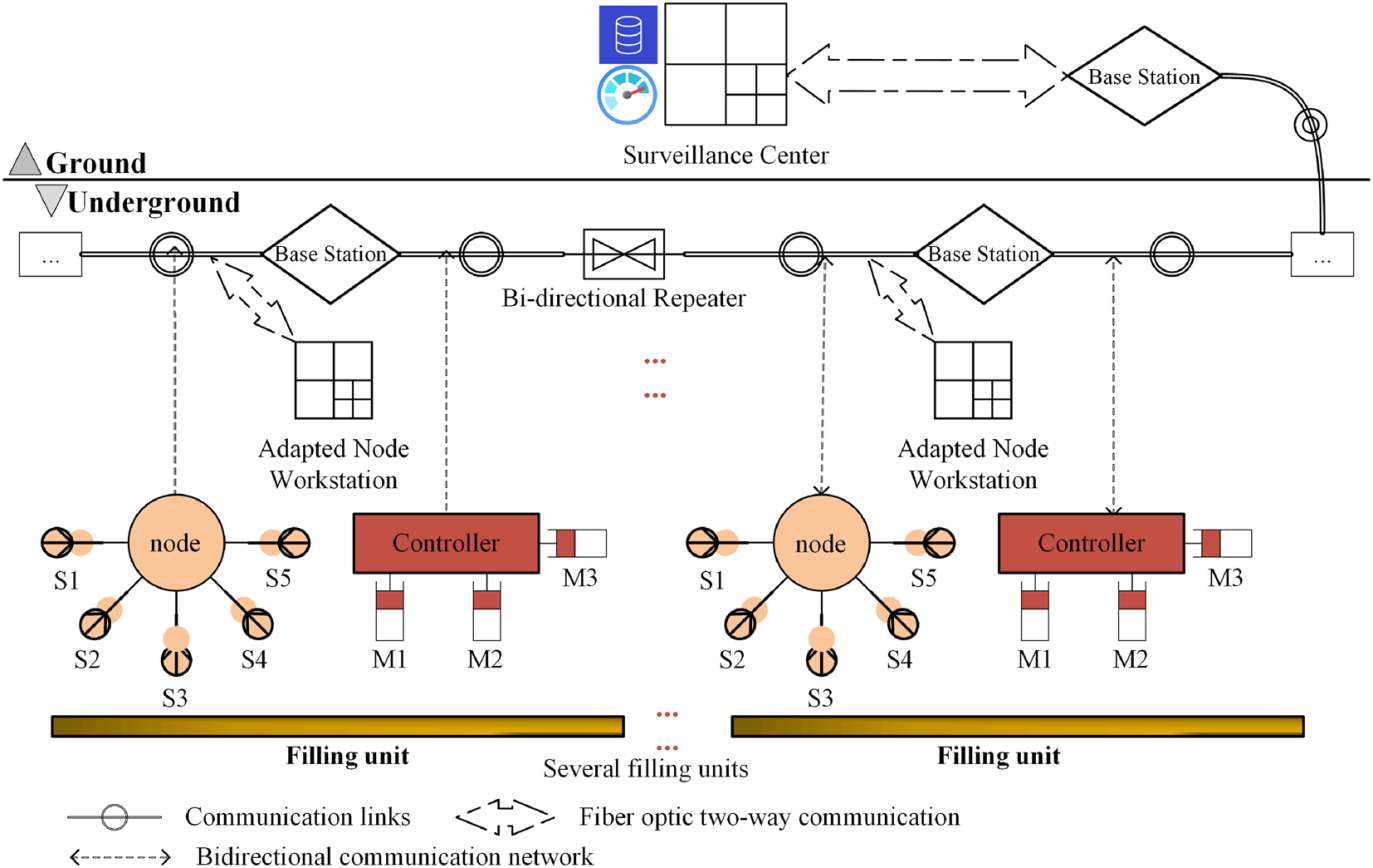
Schematic diagram of the backfilling perception Internet of Things structure.

In intelligent filling, multiple filling units perform filling operations simultaneously, as shown in Fig. 5. From a spatial point of view, the intelligent filling communication network comprises a main ground network, an underground communication ring network, and a unit sensor network. The main ground network comprises ground communication base stations and links, which perform communication tasks with the underground ring network at the bottom and with the internet at the top. The underground communication ring network comprises underground explosion-proof communication base stations and underground communication links, which perform communication tasks in the underground area and communication transfer between the ground monitoring centre and underground devices. The device detection network connects detection devices with the detection layer network. It establishes stable communication between detection devices and execution units through adapter nodes to achieve an interactive connection between the underground communication ring network and the ground monitoring centre.

Using a single filling unit as an example, a sensor network consisting of pressure sensors, tilt sensors, distance sensors, cameras, and material level sensors is set up in the single filling unit. The collected data from the sensors are uploaded to the adapter node. The adapter node connects to the communication link installed in the roadway. The communication link forms a communication network with the underground communication base station and connects signals between each filling unit through front-end communication, bidirectional relay, and back-end communication. An adapter node workstation is set up to upload real-time perception data from sensor networks in this area, which serves as a data bridge between sensor networks and data service centres. Control instructions are sent to executing agencies via adapter nodes to control equipment, such as hydraulic support jacks, hydraulic support control valve groups, and hydraulic support piping.

The ground monitoring centre establishes two-way communication with ground communication base stations. It connects to back-end communication through its communication links to establish data connections between itself and various adapter node workstations. The ground monitoring centre has the highest monitoring authority; it can monitor filling equipment operation in real time, monitor the filling environment and evaluate the filling effects; it can also perform cloud data backup and issue scheduling commands to control the operating status of the filling equipment. The ground monitoring centre consists of databases, development consoles, and monitors, where databases store standardized unified data that serve as sources for all applications; consoles provide hardware platforms for various intelligent applications such as data service centres and big data computing visualized clean-up. They can also perform intelligent analysis on filling conditions or make scientific decisions on filling actions by generating and issuing control instructions so that adjustments can be made to underground equipment operation accordingly; monitors provide visualization for intelligent filling work while providing a judgement basis for safe equipment operation.

## Research on the validity of backfilling perception system data

Ensuring that data collected by multiple sensors are up-to-date and effective requires anomaly detection. This paper proposes an anomaly detection model based on solid backfilling perception system data, which is divided into three parts: a position encoding module, a time series prediction module, and an anomaly judgement module. Based on timestamp information, the position encoding module extracts global temporal information, including year, month, and day. The time series prediction module uses a multivariate time series prediction model based on the temporal fusion transformer^23^, which not only considers potential correlations between different variables but also accounts for static covariates in the environment to predict target variables. The main task of the anomaly evaluation module is to find an appropriate threshold for error prediction and to dynamically calculate the current optimal threshold using a dynamic thresholding method.

### Position encoding

**Figure 6:**
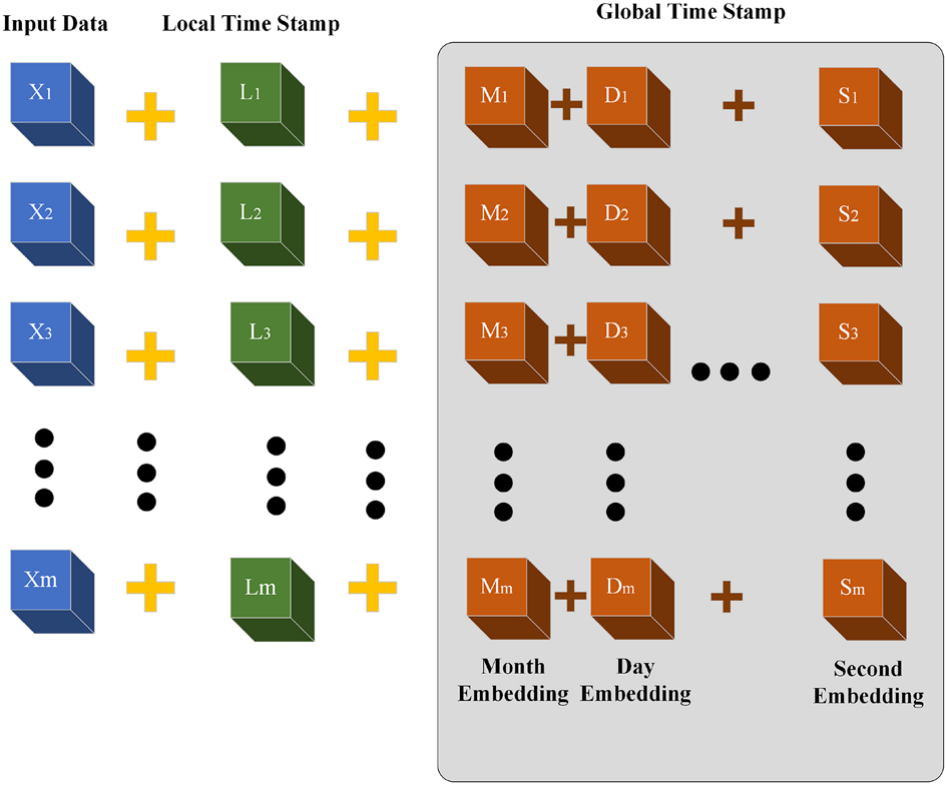
Position coding module structure diagram.

The transformer model differs from RNN models in using self-attention mechanisms to process all input data in parallel^30^. However, this approach can result in inherent positional and temporal information loss in the sequence^22^. To overcome this limitation, the original transformer model uses a local position coding method that can only encode sequences within a specific time window. Unfortunately, this method cannot extract global position information or periodic variation information present in timestamps^31^. To address these issues and to make transformer-based models more suitable for anomaly detection in multisensor systems, a new global time series encoding method based on data from solid-state sensor systems was proposed. The position encoding module is illustrated in Fig. 6.

This method adds global position coding based on timestamp information to the traditional local coding method. The year (y), month (m), day (d), hour (h), minute (m), and second (s) information in the timestamp is extracted and mapped to a specific numeric value by a function so that global position information can be obtained. Assuming that the time stamp at time t is *date*, this coding method can be used to obtain the coding vector *TPE*:

1 $$\begin{aligned} T P E_t=f_1( \text{ m } )+f_2( \text{ d } )+f_3( \text{ h } )+\cdots +f_n( \text{ s}) \end{aligned}$$

Normalize the date and numeric information in the timestamp into vector elements.

2 $$\begin{aligned} \left\{ \begin{array}{*{20}l} f_1( \text{ m } )=\frac{ \text{ m } -1}{11}-0.5 \\ f_2( \text{ d } )=\frac{ \text{ d-1 } }{30}-0.5 \\ f_3( \text{ h } )=\frac{ \text{ h } }{23}-0.5 \\ \vdots \\ f_n( \text{ s } )=\frac{ \text{ s } }{59}-0.5 \\ ( \text{ m, } \text{ d, } \text{ h } , \cdots , \text{ s } ) \in \text{ d } \end{array}\right. \end{aligned}$$

Within this group, $f_i$ represents the encoding formula corresponding to scaling all time information to the interval $[-0.5, 0.5]$. Each timestamp is then encoded into an individual element and combined with others to form a comprehensive global time sequence vector. This vector is then appended to the input data, along with the local position coding vector, as a sequence that accurately reflects temporal features.

### Time series prediction model

In the actual backfilling mining process, the factors that affect the filling effect include not only environmental variables monitored in real time by sensor networks but also some important static factors such as fill material ratio, gangue aggregate grading, compaction distance from the top and drop centre distance. These static factors also have potential effects on other variables. Therefore, considering static factors is crucial to improving target sequence prediction accuracy. In addition, the filling operation has a periodicity. Different stages of sensor data changes have different patterns during the entire work cycle. Periodic changes may be reflected in known future input sequences, such as work time information. Therefore, a time series prediction model should have functions to process and utilize different types of input sequences (static covariates, future deterministic input sequences, and future uncertain input sequences) differently.

In addition, the sensor network on the filling surface contains at least dozens of sensors. When predicting the target sequence, the degree of correlation between other sensor sequences and the target sequence varies greatly; some are highly correlated, while others may be irrelevant. Therefore, the model must have a feature selection function to select highly correlated sensor sequences as feature variables while suppressing irrelevant sequences during the modelling phase.

**Figure 7:**
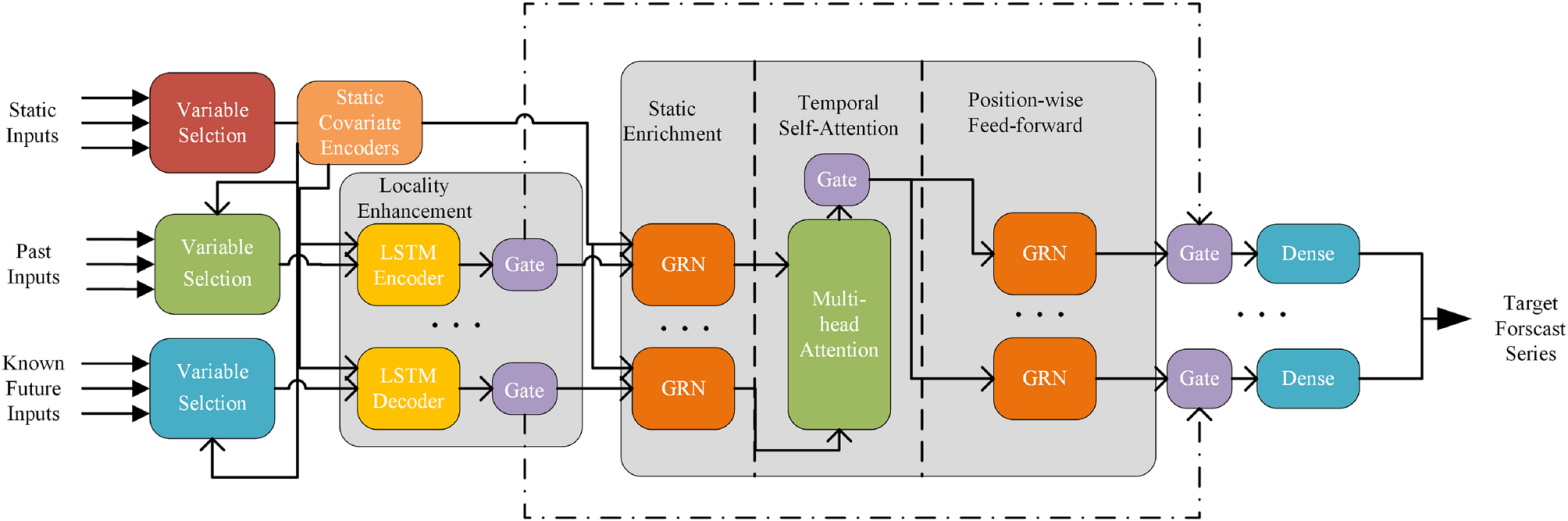
Time series prediction model structure diagram.

The transformer-based time series prediction model for solid fill perception system data is based on the transformer variant shown in Fig. 7. The model uses a self-attention mechanism as its core and sends different sequences to different model modules. It also includes a static covariate encoder and a variable selection network to improve prediction performance. The static covariate encoder can encode covariates into context vectors to enhance temporal features; the variable selection network can select important sequences from input sequences, reduce the impact of irrelevant sequences on predictions, and improve modelling performance for time series.

#### Variable selection network

To suppress the irrelevant sequences in the input multivariate time series that are unrelated to the target variable and to ensure the flow of effective information, we first introduce a gated residual network (GRN) whose inputs are the vector *a* and an optional external context vector *c*:

3 $$\begin{aligned}{} & {} {\text {GRN}}_\omega (a, c)={\text {LayerNorm}}\left( a+{\text {GLU}}_\omega \left( \eta _1\right) \right) \end{aligned}$$

4 $$\begin{aligned}{} & {} \eta _1=W_{1, \omega } \eta _2+b_{1, \omega } \end{aligned}$$

5 $$\begin{aligned}{} & {} \eta _2={\text {ELU}}\left( W_{2, \omega } a+W_{3, \omega } c+b_{2, \omega }\right) \end{aligned}$$

6 $$\begin{aligned}{} & {} {\text {GLU}}_\omega (\gamma )=\sigma \left( W_{4, \omega } \gamma +b_{4, \omega }\right) \odot \left( W_{5, \omega } \gamma +b_{5, \omega }\right) \end{aligned}$$

In the formula, ELU is the exponential linear activation unit function, $\eta _1$ and $\eta _2$ are intermediate layers, LayerNorm is a standard normalization layer, and $\omega$ represents the weighting. GLU is a gated linear unit that can suppress unnecessary parts of the dataset.

In each prediction step, all time series are entered into the model. However, the correlation between different time series and the target time series is unknown and different. Time series with high correlation have higher contributions to the prediction of target sequences, while those with low correlation have lower contributions or may become noise that reduces prediction accuracy. Therefore, to improve prediction accuracy, we use variable selection networks to assign weights based on the correlation between time series and target sequences before inputting weighted data into the model for prediction.

A total of three variable selection nets are set up in the model with inputs of static time series, past series, and future series. The parameters of different variable selection nets are not shared but have the same structure. Let $X_t^j$ denote the past time series generated by the *j*th sensor at time *t*, and all past time series $\Xi _t=[(X_t^1)^T,\dots , (X_t^{m_\chi })^T]^T$ at time *t* and the context vector $c_s$ are input to the GRN layer in Eq. (3,4,5,6) and then passed through the softmax layer, resulting in a $m_\chi$ dimensional weight vector:

7 $$\begin{aligned} v_{\chi t}={\text {Softmax}}\left( {\text {GRN}}_v\left( \Xi _t, c_s\right) \right) \end{aligned}$$

At the same time, each time series $X_t^j$ is entered into a GRN layer for nonlinear processing:

8 $$\begin{aligned} \widetilde{X_t^j}=\textrm{GRN}_{X^j}\left( X_t^j\right) \end{aligned}$$

Each time series $X^j$ has its own GRN layer, and its parameters are shared across all time steps. Finally, all feature vectors $(X_t^j)^\sim$ processed by the GRN are multiplied by the weight vector $v_{\chi t}$ to obtain a weighted time series, which plays a role in variable selection.

9 $$\begin{aligned} \widetilde{X_t}= \sum _ {j = 1} ^ {m_\chi } v_ { \ chi t } ^ j \widetilde{ X _t ^ j } \end{aligned}$$

#### Static covariate encoder

During the filling and mining process, there are some static environmental parameters, such as the distance from the compacted top and the distance from the centre of the material. Considering these static covariates during prediction can improve prediction accuracy. Therefore, a static covariate encoder is set up in the model to integrate information about static variables and improve the predictive ability of the model. The static covariate encoder uses four different GRNs to generate four different context vectors $c_s,c_c,c_e,c_h$, where $c_s$ inputs the variable selection network, $c_c,c_h$ inputs the long short-term network (LSTM) for local processing of time features, and $c_e$ inputs a static enhancement layer to enrich time features. For example, in the variable selection network, the context vector $c_s$ is generated by the GRN network through a static time series X.

10 $$\begin{aligned} c_s=GRN_{c_s}(X) \end{aligned}$$

#### Time-domain fusion encoder

##### End-to-end local enhancement layer

Prior to utilizing the time-domain fusion encoder, it is necessary to establish a local enhancement layer that will improve the local characteristics of the time series. This layer operates end-to-end and employs an LSTM encoder to process past time series $X_{t-k:k}$, and an LSTM decoder for future known time series $X_{t+1:t+\tau _{max}}$. The output from both these components generates a unified sequence feature that serves as input for the time-domain fusion encoder. This feature is denoted by $\phi (t,n) \in {\phi (t,-k),\dots ,\phi (t,\tau _{max})}$, where *n* represents its position index. Prior to entering the fusion encoder, $\phi (t,n)$ undergoes the GLU operation once again for variable selection.

11 $$\begin{aligned} {\tilde{\phi }}(t, n)={\text {LayerNorm}}\left( {\tilde{X}}_{t+n}+{\text {GLU}}_{{\widetilde{\phi }}}(\phi (t, n))\right. \end{aligned}$$

##### Static enhancement layer

A static enhancement layer is set in the time-domain fusion encoder to better utilize static covariates to enhance the static temporal data features. The outputs of the local enhancement layers $\phi (t,n)$ and $c_e$ generated by the static covariate encoder are input together into the static enhancement layer.

12 $$\begin{aligned} \theta (t,n)=\text {GRN}_{\theta }(\phi (t,n),c_e) \end{aligned}$$

##### Temporal self-attention layer

After passing through the static enhancement layer, all $\theta (t,n)$ generated by the data are flattened to obtain a matrix $\Theta (t)=[\theta (t,-k),\cdots ,\theta (t,\tau )]^T$. The self-attention mechanism can learn long-term dependencies in temporal data, and multihead self-attention can further improve model performance by allowing the model to focus on different information aspects.

13 $$\begin{aligned} B(t)={\text {Multihead}}(\Theta (t), \Theta (t), \Theta (t)) \end{aligned}$$

In the formula, $B(t)=[\beta (t,-k),\cdots ,\beta (t,\tau _{max})]$. We added a decoder mask mechanism in the self-attention layer to prevent future sequence information from leaking into the past during training. We again added a gating layer to enhance the variable selection in the temporal self-attention layer:

14 $$\begin{aligned} \delta (t, n)={\text {LayerNorm}}\left( \theta (t, n)+{\text {GLU}}_\delta (\beta (t, n))\right) \end{aligned}$$

##### Positional feedforward layer

The positional feedforward layer performs additional nonlinear processing on the output of the self-attention layer and selects the output again.

15 $$\begin{aligned} \psi (t, n)={\text {GRN}}_\psi (\delta (t, n)) \end{aligned}$$

In addition, to alleviate gradient vanishing and network degradation, we introduce residual connections that skip the entire temporal fusion decoder:

16 $$\begin{aligned} {\tilde{\psi }}(t, n)={\text {LayerNorm}}\left( {\tilde{\phi }}(t, n)+{\text {GRN}}_{{\tilde{\psi }}}(\delta (t, n))\right) \end{aligned}$$

### Anomaly detection

**Figure 8:**
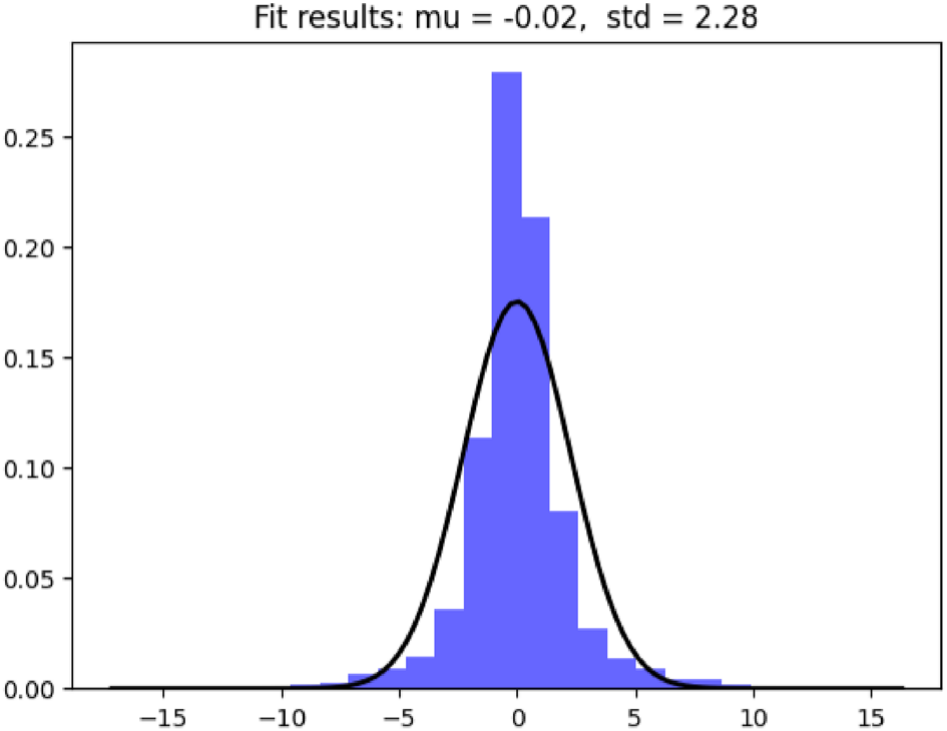
Predictive error distribution diagram.

When training a time series prediction model, we use a dataset that contains only normal data. Therefore, the model learns only the spatiotemporal relationship of normal data, and its output prediction values have small errors compared to the actual values under normal circumstances. If there is a large error between the predicted value and the actual value, it can be considered abnormal point data. Therefore, the anomaly detection module needs to calculate the prediction error of the time series prediction module and determine whether the actual value deviates from the normal range based on this error. As shown in Fig. 8, the prediction error for normal data follows a Gaussian distribution. Therefore, we calculate the mean and variance in the prediction errors during the training phase. Then, we determine the threshold for judgement according to the $3\sigma$ criterion in statistics. Based on the relationship between the prediction error and the fixed threshold, we judge whether the actual values are anomalies: if the prediction error is greater than the fixed threshold, it is judged that the actual values deviate from the normal range and belong to anomalous points; otherwise, they are considered normal values.

In the real-time anomaly detection phase, we first construct a sliding window *W* with a length of *h*. At each moment *t*, the data in the sliding window are:

17 $$\begin{aligned} W=\left[ y^{(t-h+1)}, \ldots , y^{t-1}, y^t\right] \end{aligned}$$

In the formula, $y^{\left( t\right) }$ represents the true value at time *t*. The time series prediction module predicts the value at time $t+1$ by using data within a sliding window at time *t*, generating a predicted value ${\hat{y}}^{\left( t+1\right) }$. After the arrival of time $t+1$, the anomaly detector obtains the actual value at time $t+1$ and calculates the prediction error for that moment.

18 $$\begin{aligned} e^{t+1}=\left| y^{(t+1)}-{\hat{y}}^{(t+1)}\right| \end{aligned}$$

**Figure 9:**
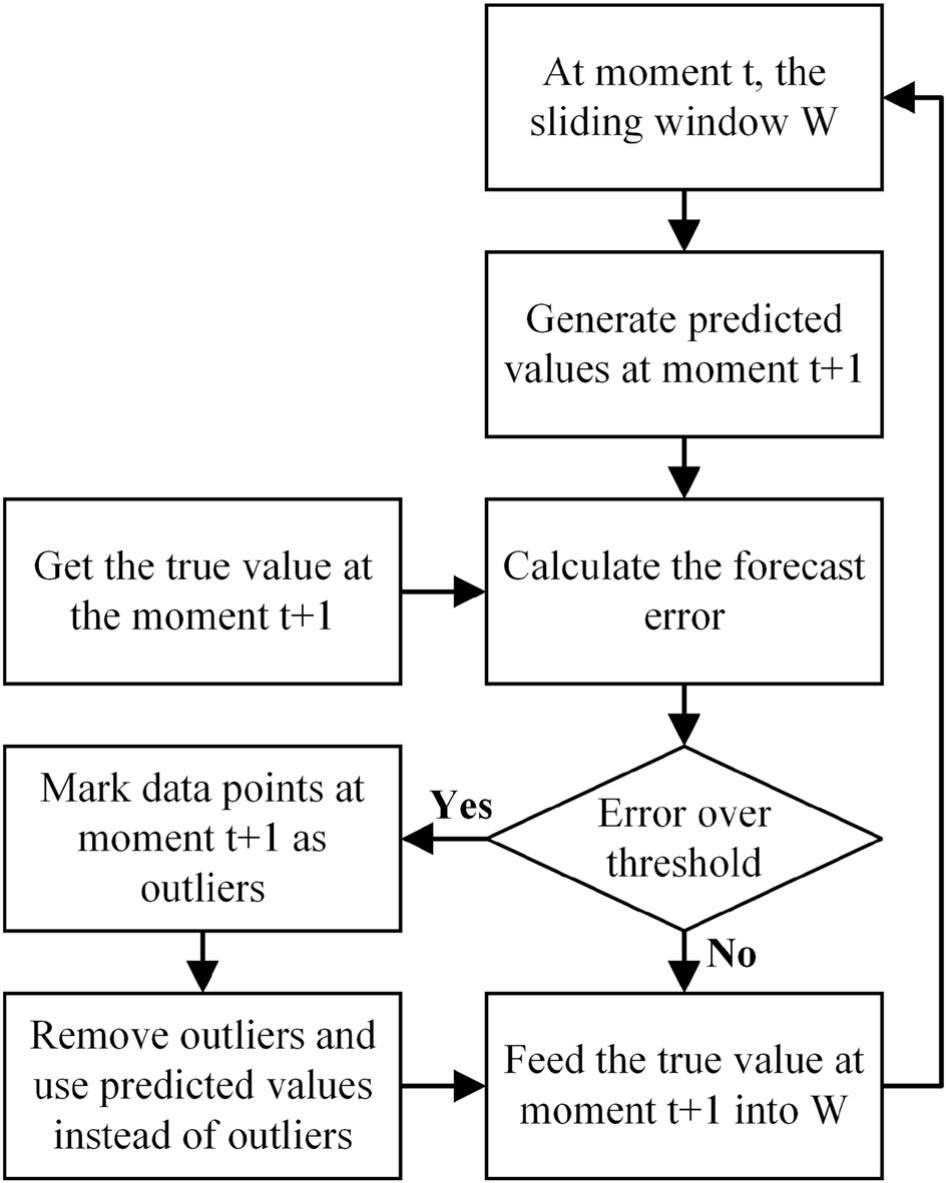
Abnormal judgement flowchart.

In the formula, $y^{(t)}$ represents the true value at time *t*. The time series prediction module predicts the value at time $t+1$ by using data within a sliding window at time *t*, generating a predicted value ${\hat{y}}^{(t+1)}$. Upon arrival at time $t+1$, the anomaly detector obtains the actual value and calculates the prediction error for time $t+1$. Then, the model compares $e^{(t+1)}$ with the threshold value $\epsilon$ we set. When $e^{(t+1)}$ is less than $\epsilon$, the data point at that time is judged as a normal point, and $y^{(t+1)}$ is sent to the sliding window; otherwise, the data point at that time is judged as an abnormal point, removed from the system, and interpolated with predicted value ${\hat{y}}^{(t+1)}$ to fill in missing values at time $t+1$. The resulting ${\hat{y}}^{(t+1)}$ is then sent to the sliding window. Figure 9 shows the process for judging anomalies.

## Experimental verification

### Dataset and performance metrics

We conducted experiments on a real dataset to verify that our proposed model can be applied to the sensor network of an intelligent filling workface. The real dataset was collected from a sensor network of a filling workface. Due to the large number of sensors in the filling workface and high sampling frequency, massive data are generated. Therefore, we selected data from August 1st to August 31st, 2022, for training and testing the model, which includes 15 parameters. The data for training the prediction models contain 50,000 records with expert-removed and interpolated abnormal data. The test set contains 1200 records with expert-labelled abnormal points.

Although the anomaly detection model proposed in this article includes a data prediction module, its ultimate task is determining whether a data point is an outlier. Therefore, this problem essentially belongs to a binary classification problem. We define outliers as the positive class and normal points as the negative class. We use *TP* to represent the number of data points predicted by the model as outliers, *TN* represents the number of data points predicted by the model as normal points, *FP* represents the number of normal points predicted by the model as outliers and *FN* represents the number of outliers predicted by the model as normal points. Considering that outlier values are much fewer than normal values, common indicators such as the accuracy rate, recall rate, missed detection rate and false alarm rate were selected when measuring the performance of this model:

19 $$\begin{aligned} \left\{ \begin{array}{l} P r e c=\frac{T P}{T P+F P} \\ {\text {Rec}}=\frac{T P}{T P+F N} \\ F A R=\frac{F P}{F P+T N} \\ M A R=\frac{F N}{F N+T P} \\ F 1=\frac{2 \times \text{ Prec } \times \text{ Rec } }{ \text{ Prec } + \text{ Rec } }=\frac{2 \times T P}{2 \times T P+F P+F N} \end{array}\right. \end{aligned}$$

In the formula, $\text {Prec}$ represents precision, $\text {Rec}$ represents recall, and $\text {F1}$ represents the F1 score that comprehensively considers both precision and recall indicators. The higher these three indicators are, the better the model performance; $\text {FAR}$ represents the false alarm rate, and $\text {MAR}$ represents the missed alarm rate. The lower these two indicators are, the better the model performance.

#### Experimental results and analysis

The authors selected 4 variables from the dataset for prediction. We selected data generated by four different types of sensors for conducting tests on anomaly detection. In the subsequent text, these sensors included a conveyor speed sensor, feeding system weight sensor, material drop height sensor, and compaction mechanism stroke sensor, referred to as Sensor 1, Sensor 2, Sensor 3, and Sensor 4, respectively. They were deployed in the feeding and conveying systems, and filling face of the backfill mining system to monitor various types of physical measures. Due to the structural and measurement differences among these sensors, the data from these four sensors exhibit distinct variation patterns, reflecting the multisource heterogeneity of the sensor network. First, the variable to be detected for anomalies was set as the target variable for prediction. Other variables and parameters were used as covariates, and a time series prediction model was trained. In addition, the threshold for anomaly detection was determined based on the distribution of prediction errors. After completing training, we input test datasets containing abnormal points into the prediction module and input the output results into an anomaly detection module. The existence of abnormal points was judged by calculating an anomaly score.

**Figure 10:**
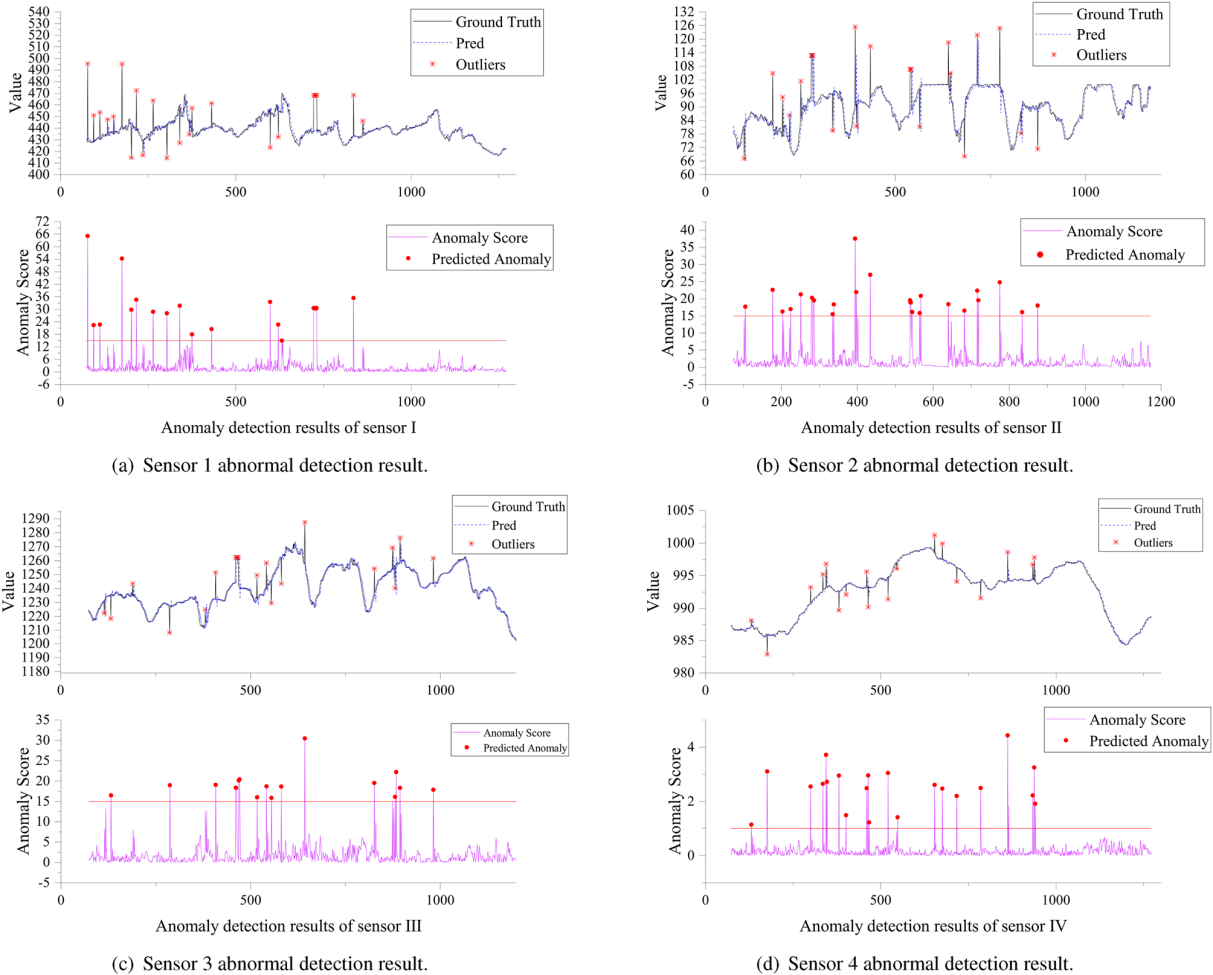
Abnormal sensor detection results.

Figure 10 shows the final abnormal detection results. The upper and lower parts of the figure represent the sensor values, the black line represents the actual output value of the sensor, and the blue line represents the predicted value generated by the prediction module. The lower part shows the anomaly score for each time point, representing the difference between the predicted and actual values. The higher this score is, the more likely it is that this point is an anomaly. The points marked with “*” on the actual value curve are anomalies marked by experts. Red circular points on the anomaly score graph are anomalies determined by the model based on predicted values. The red line is a decision threshold determined in this paper based on the normal point prediction error distribution and practical experience; our model identified points above this threshold. These figures show good agreement between the actual anomalous points and those detected by our model, indicating that our proposed abnormal detection model is highly effective when applied to filling mining datasets.

Figure 10a shows that there are 30 abnormal data points in the sensor 1 data, among which 25 abnormal data points were identified with a recognition rate of 83.33%. Figure 10b shows that there were 25 abnormal data points in the sensor 2 data, among which 22 abnormal points were identified with a recognition rate of 88.00%. Figure 10c shows that there were 21 abnormal data points in the sensor 3 data, among which 17 abnormal points were identified with a recognition rate of 80.95%. Figure 10d shows that there were 19 abnormal data points in the sensor 4 data, and 18 abnormal points were identified with a recognition rate of 94.74%. This indicates that the model performs well.

**Figure 11:**
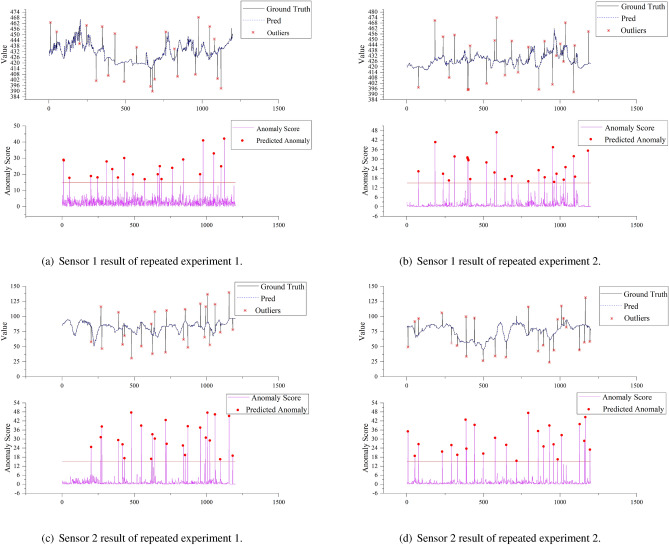
Model repeatability and robustness verification results of sensor1 and sensor2.

**Figure 12:**
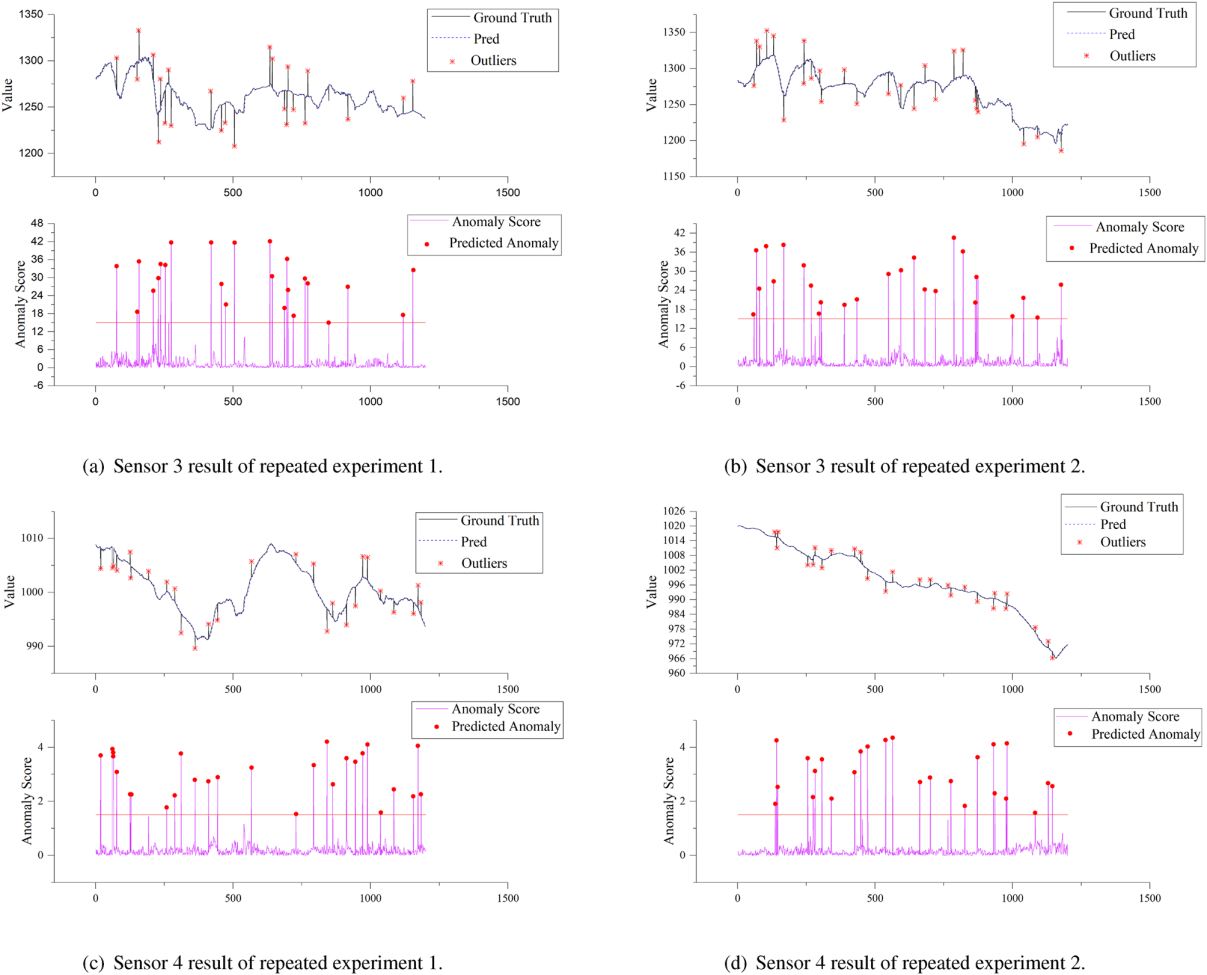
Model repeatability and robustness verification results of sensor3 and sensor4.

**Table 2:** Results of abnormal detection for each sensor.

Sensors	Experiments	Prec (%)	Rec (%)	F1-score	FAR (%)	MAR (%)
Sensor 1	1	96.00	83.33	0.89	0.085	16.67
	2	92.59	89.29	0.91	0.17	10.71
	3	96.32	92.96	0.95	0.83	7.12
	Averge	94.97	88.53	0.92	0.36	11.50
Sensor 2	1	88.00	88.00	0.88	0.25	12.00
	2	96.00	92.31	0.94	0.83	7.76
	3	93.33	90.32	0.92	0.17	9.68
	Averge	92.44	90.21	0.91	0.42	9.81
Sensor 3	1	100.00	80.95	0.89	0.00	19.05
	2	89.66	92.86	0.91	0.25	7.14
	3	88.89	96.21	0.92	0.25	4.00
	Averge	92.85	90.01	0.91	0.17	10.06
Sensor 4	1	85.71	94.74	0.90	0.25	5.26
	2	90.31	93.37	0.93	0.31	6.74
	3	87.02	96.43	0.91	0.82	3.61
	Averge	87.68	94.85	0.91	0.46	5.20

To enhance the robustness and reliability of our proposed anomaly detection model for general application, additional tests were conducted on each type of sensor data. In order to validate and strengthen the model, two additional tests were performed for each selected type of sensor, in addition to the initial test mentioned earlier in this study. Figures 11 and 12 shows the additional abnormal detection results. A more in-depth analysis of the anomaly detection results is presented in Table 2. It can be seen that the model proposed in this paper achieves high accuracy, with an accuracy rate of up to 100% for anomaly detection on sensor 3. The average accuracy rate up to 94.97% on sensor 1, indicating that approximately 90% of the abnormal points identified by our proposed model were actual anomalies. The averge recall rate of the model was as high as 94.85% on sensor 4, with an average recall rate of 90.90% among all sensors. This indicates that approximately 90% of actual anomalies were detected by the model. The model had a very low false positive rate, with an average false positive rate as low as 0.35%. This means that only approximately 0.35% of normal points were incorrectly identified as anomalies by the model. The missed alarm rate was also relatively low, with an average missed alarm rate of only 9.14%, meaning that only approximately 10% of anomalies were not detected by the system.

Furthermore, the F1 scores for different types of sensors were similar and ranged from 0.91 to 0.92, indicating good generalization performance across different types of sensor data. This indicates that the anomaly detection model demonstrated good and similar performance across these four diverse and heterogeneous sensors, suggesting its strong generalization capability. Its performance is not dependent on specific sensor data types or patterns of variation. Additionally, with ample training data, our model achieves end-to-end data processing. It analyses sensor data from only a time series perspective, autonomously learning and extracting data features without manually setting prior information regarding sensor types or variation trends.

The results obtained from anomaly detection can serve as reference factors for checking sensor stability and optimizing experimental platforms when combined with time and workstation information where abnormal data occur.

## Conclusion

This article proposes a coal mine-filling material Internet of Things (IoT) perception network framework to address the shortcomings in perception and intelligence faced by solid backfilling in coal mines. The framework can collect, transmit, process, and store sensor data during the solid backfilling process in coal mines. It also integrates data from various links into a unified data centre for rapid centralized management. The data validity under this framework and its effectiveness in expressing system information were studied, including aspects such as accuracy, reliability, timeliness, consistency and completeness, to ensure data quality and effectiveness. Experimental verification shows that the proposed method has high precision, good stability and strong scalability advantages that can effectively solve problems such as missing sensing data, low fusion degree and isolated effect of data islands.

## Data Availability

The datasets generated and/or analysed during the current study are available from the corresponding author upon reasonable request.
